# Tinzaparin Provides Lower Lipid Profiles in Maintenance Hemodialysis Patients: A Cross-Sectional Observational Study

**DOI:** 10.1155/2014/486781

**Published:** 2014-11-13

**Authors:** Ming-Hsien Tsai, Yu-Wei Fang, Jyh-Gang Leu

**Affiliations:** ^1^Division of Nephrology, Department of Internal Medicine, Shin-Kong Wu Ho-Su Memorial Hospital, No. 95 Wen-Chang Road, Shih-Lin District, Taipei 111, Taiwan; ^2^Institute of Epidemiology and Preventive Medicine, College of Public Health, National Taiwan University, No. 17 Hsu Chow Road, Zhongzheng District, Taipei 100, Taiwan; ^3^Fu-Jen Catholic University School of Medicine, No. 510 Zhongzheng Road, Xinzhuang District, New Taipei City 24205, Taiwan

## Abstract

As a low-molecular-weight heparin, tinzaparin has effectively been used as an anticoagulant during hemodialysis sessions. However, the impact of different heparin types on dyslipidemia is still controversial. In our study, 434 chronic hemodialysis patients were evaluated. The mean age was 65 ± 13. Forty-eight patients (11%) and 386 patients (89%) were in the tinzaparin and unfractionated heparin (UFH) groups, respectively. Triglyceride had significant difference between the two groups (*P* = 0.001) but total cholesterol, HDL, or LDL did not. In the univariate analysis, the triglyceride level was significantly associated with tinzaparin use [*β*: −39.9, 95% confidence interval (CI): −76.7 to −3.0], and this association remained following the multivariate analysis (*β*: −40.8, 95% CI: −75.1 to −6.5). The difference in serum total cholesterol level between tinzaparin and UFH became significant (*β*: −13, 95% CI: −24.5 to −1.56) after adjustment in the multivariate analysis. Moreover, in a subgroup analysis, male diabetic patients showed lower serum triglyceride levels with the use of tinzaparin, while older, nondiabetic, male patients showed significant advantages in total cholesterol levels with the use of tinzaparin. Based on our findings, tinzaparin shows a significant association with a lower lipid profile in patients with chronic hemodialysis when compared to UFH.

## 1. Background

An anticoagulant is needed during hemodialysis (HD) to prevent activation of the blood coagulation system and the subsequent fibrin clot formation and platelet aggregation, which results in dialyzer dysfunction and widespread clotting of the tubing. Although unfractionated heparin (UFH) has been used routinely since the late 1930s, low-molecular-weight heparin (LMWH) is increasingly being used in the current practice of HD [1]. UFH has a number of disadvantages, including bleeding, thrombosis [2], osteoporosis [3], thrombocytopenia [4], and lipid abnormalities [5]. An important reason underlying the shift in heparin use is that LMWH is reported to have potential advantages over UFH, such as a low risk of heparin-induced thrombocytopenia [5] and a favorable lipid profile [6].

Dyslipidemia is a risk factor for cardiovascular disease, one of the major leading causes of mortality in patients with chronic HD. Lipoprotein lipase (LPL) is an extrahepatic enzyme that controls the intravascular delipidation of chylomicrons and very-low-density lipoproteins, and UFH affects fat metabolism through its capacity to mediate the release of hepatic lipase and LPL from the vascular endothelium into the blood stream [7]. Repeated administration of heparin for anticoagulation during HD can cause the depletion of LPL and may exhaust lipolytic capacity, resulting in the slowed metabolism of triglyceride- (TG-) rich lipoproteins [8, 9]. However, the question of whether LMWH stimulates plasma lipase to the same extent as UFH is unresolved [10, 11], and its effect on uremic dyslipidemia is also undetermined. Moreover, although several studies have compared a variety of LMWHs with UFH in HD patients, the results were controversial [6, 12–23]. Further, of these LMWHs, the experience with tinzaparin sodium (Innohep, Leo Pharmaceutical Corp.) is limited [12, 15, 24]. Badawi reported decreased total cholesterol (TC) but increased TG during the administration of LMWH over a 3-month period in 30 HD patients [15]. Meanwhile, Sabry et al. [24] and Al-Saran et al. [12] found no observed benefit on lipid profiles in 23 HD patients after shifting to LMWH during a 6-month period. Therefore, the study aimed to clarify the hypothesis that tinzaparin may lead to a better lipid profile through comparing the clinical lipid profiles between chronic HD patients receiving tinzaparin or UFH.

## 2. Methods

### 2.1. Study Population

Four hundred thirty-four patients with regular HD were analyzed from outpatient nephrology clinics at Shin-Kong Wu Ho-Su Memorial Hospital, Taipei, Taiwan, in 2012. Eligibility requirements were being at the age of ≥30 and receiving regular HD in our hospital. Exclusion criteria included HD less than 6 months, known hypersensitivity to UFH or tinzaparin, family hyperlipidemia, malignancy, and ongoing inclusion in another study. UFH was given intravenously at a dose of 20–35 unit/kg to prevent artificial kidney coagulation. Tinzaparin (a LMWH) was also given intravenously at a dose of 1000–3000 units according to the patient's coagulation status with an artificial kidney. The Institutional Review Boards of the Shin-Kong Wu Ho-Su Memorial Hospital, Taipei, Taiwan, approved the study.

### 2.2. Data Collection

Demographic data and medical data were collected, which included age, gender, body mass index (BMI), HD time, the presence of diabetes mellitus, blood pressure, prescription of lipid-lowering agents, and the types of artificial kidney. Moreover, the laboratory data were obtained from medical charts, including hemoglobin (Hb), TG, TC, high-density lipid (HDL), low-density lipid (LDL), aspartate aminotransferase (AST), alanine aminotransferase (ALT), alkaline phosphatase (Alk-p), albumin, intact parathyroid hormone (iPTH), iron profile, total-bilirubin (T-bili), uric acid (UA), sodium (Na), potassium (K), ion-calcium (iCa), and phosphate (P). Dialysis efficiency was evaluated according to the Kidney Disease Outcomes Quality Initiative guidelines, and the single-pool Kt/V of urea nitrogen was calculated [25]. Blood samples were drawn before dialysis and after at least 8 hours of fasting.

### 2.3. Statistical Analyses

This study is designed as a retrospective cross-sectional study. Data are expressed as the mean ± SD and the range or frequency, as appropriate. For analytical purposes, patients were divided according to the use of tinzaparin or UFH. Intergroup comparisons were performed using a *χ* ^2^ test for categorical variables, and the independent *t*-test was used for continuous variables. Subsequently, we performed a multivariate linear regression analysis to investigate the potential factors independently associated with tinzaparin use and components of the lipid profile. All factors in the crude analysis were put into a forward stepwise multivariable analysis. Further, we included tinzaparin use as the primary predictor for serum TG or TC levels in the following subgroups: DM, non-DM, female, male, age of <60, and age of ≥60. The association was adjusted for those factors selected by the forward stepwise multivariable analysis. A 2-sided *P* < 0.05 was considered statistically significant. All statistical analyses were performed with the statistical package for Social Sciences Statistical Software (SPSS version 20; IBM, Chicago, IL).

## 3. Results

### 3.1. Patient Characteristics

There were 434 chronic HD patients (210 males and 224 females) with an average age of 65.0 ± 13.2, a mean BMI of 22.6 ± 3.7 kg/m^2^, and HD duration time of 7.7 ± 5.6 years. Of these patients, 177 (40.8%) had diabetes and 68 (15.7%) were under antilipidemic medication control. The mean systolic blood pressure (SBP) and diastolic blood pressure (DBP) were 142 ± 25 mmHg and 80 ± 62 mmHg, respectively. The lipid profile of the entire study population had a mean TC of 172.4 ± 41.1 mg/dL (coefficient of variation (CV) = 23.83%), a mean TG of 141.9 ± 122.9 mg/dL (CV = 86.60%), a mean HDL of 50.6 ± 19.1 mg/dL (CV = 37.74%), and a mean LDL of 105.2 ± 35.7 mg/dL (CV = 33.93%). Moreover, in terms of HD adequacy, the kt/v was 1.39 ± 0.19; Hb, 10.3 ± 1.26 g/dL; albumin, 4.2 ± 0.4 g/dL; iPTH, 261 ± 304 pg/mL; iCa, 4.93 ± 0.5 mg/L; and P, 5.1 ± 1.4 mg/dL. The types of artificial kidney used were polysulfone (61.5%), polymethylmethacrylate (27.9%), and cellulose citrate (10.6%).

Table 1 lists the demographic and clinical data for the 434 participants as stratified by tinzaparin and UFH use. Among the 434 participants, 48 (11%) and 386 (89%) received tinzaparin and UFH, respectively. Those receiving tinzaparin had significantly lower ferritin (*P* = 0.044) and total bilirubin levels (*P* < 0.001) and showed higher HD vintage (*P* < 0.001), Na (*P* = 0.017), and iCa levels (*P* < 0.023).

**Figure 1:**
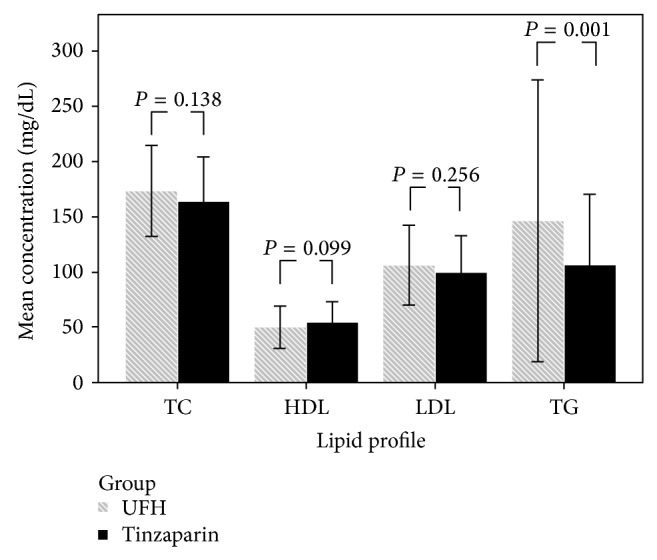
Differences in the lipid profiles between patients receiving conventional unfractionated heparin (UFH) or tinzaparin. Triglyceride was significantly different between the two groups (*P* = 0.001), but total cholesterol (TC), high-density lipoprotein (HDL), and low-density lipoprotein (LDL) showed no significant differences.

However, the differences in age, gender, DM, blood pressure, BMI, lipid-lowering agents, type of artificial kidney, Kt/V, iPTH, Hb, albumin, AST, ALT, iron, total iron-binding capacity (TIBC), Alk-P, P, and K levels were not statistically significant between the two groups (*P* > 0.05 for all).  Figure 1 shows the lipid profiles of the two groups. Significant differences were observed in the TG level (*P* = 0.001) but not in the TC (*P* = 0.138), HDL (*P* = 0.099), and LDL (*P* = 0.256) levels.

### 3.2. Multivariate Analyses of the Lipid Profiles

As shown in Tables 2 and 3, tinzaparin can significantly predict serum TG levels (*β*: −39.9, 95% CI: −76.7 to −3.0) but not serum TC (*β*: −9.3, 95% CI: −21.6 to 3.0), LDL (*β*: −6.2, 95% CI: −16.9 to 4.5), and HDL levels (*β*: 4.8, 95% CI: −0.9 to 10.5). The association between tinzaparin use and TG levels remained following multivariate adjustment (*β*: −40.8, 95% CI: −75.1 to −6.5). Furthermore, after multivariate adjustment, tinzaparin became an independent factor for TC levels (*β*: −13.0, 95% CI: −24.5 to −1.56) but not serum LDL and HDL levels.

In the crude analysis, various factors were associated with serum TG levels, including diabetes, BMI, SBP, lipid-lowering agent, HD time, Kt/V, TIBC, iPTH, sodium, and uric acid. Moreover, the male gender, age, diabetes, albumin, hemoglobin, t-bilirubin, iron, TIBC, potassium, phosphate, and uric acid showed a significant relation with serum TC levels (Table 2). Additionally, age, lipid-lowering agent, PMMA, albumin, AST, ALT, hemoglobin, total-bilirubin, ferritin, iron, TIBC, potassium, and uric acid were significantly associated with serum LDL levels, while the male gender, diabetes, BMI, HD time, Kt/V, albumin, AST, ALT, total bilirubin, ferritin, iPTH, potassium, iCa, and uric acid were related to serum HDL levels (Table 3).

In a stepwise multiple regression, tinzaparin, male gender, BMI, SBP, HD time, TIBC, sodium, phosphate, and uric acid were significantly related to serum TG levels; tinzaparin, male, diabetes, HD time, albumin, Hb, and TIBC to serum TC levels; male gender, lipid-lowering agent, AK, albumin, AST, and iron to serum LDL levels; and age, DM, BMI, kt/V, albumin, ferritin, and iPTH to serum HDL levels.

### 3.3. Subgroup Analysis of Tinzaparin Use

**Figure 2:**
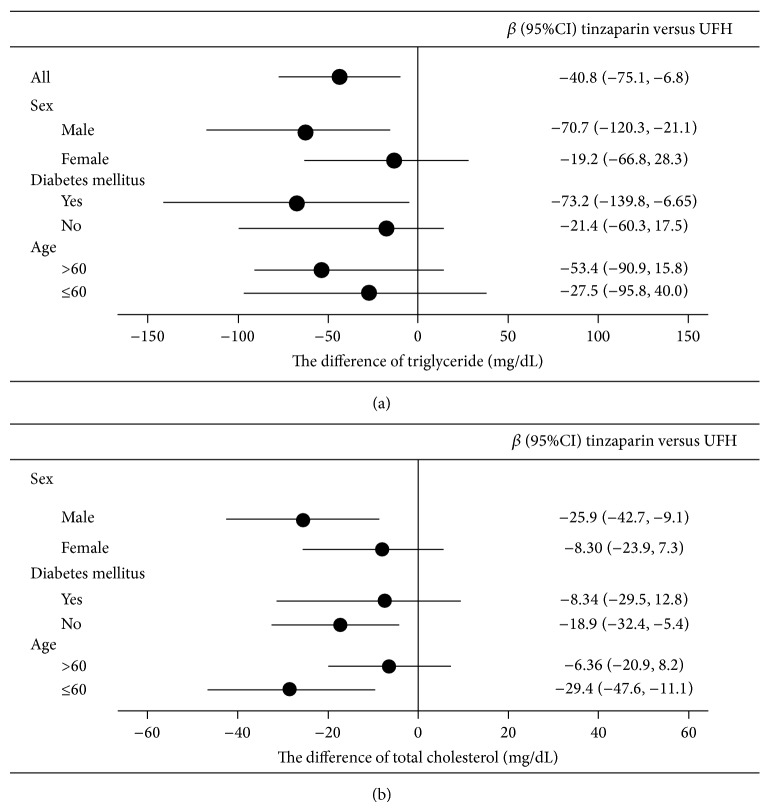
Subgroup analysis of the effect of tinzaparin on (a) serum triglyceride levels and (b) serum total cholesterol level in hemodialysis patients. The model for serum triglyceride levels was adjusted for sex, body mass index, systolic blood pressure, hemodialysis time, total iron-binding capacity, sodium, phosphate, and uric acid. The model for serum total cholesterol levels was adjusted for sex, diabetes mellitus, albumin, hemoglobin, hemodialysis time, and total iron-binding capacity.

We investigated the association between tinzaparin use and serum TC or TG levels by performing analyses in which the patients were stratified according to covariates, including the history of DM, age (>60 and ≤60 years of age), and gender. Following multivariate adjustment, the results showed that male HD patients with a history of DM had lower serum TG levels with the use of tinzaparin than UFH (Figure 2(a)) and that male non-DM patients younger than 60 years of age showed significantly lower serum TC levels with the use of tinzaparin than UFH (Figure 2(b)).

## 4. Discussion

In this cross-sectional study of 434 participants with chronic HD, those with tinzaparin use had lower serum TG and TC levels than those with UFH use. The effect of tinzaparin on lipid profiles in HD patients was independent of traditional hyperlipidemia risk factors, including gender, age, BMI, DM, artificial kidney, blood pressure, and HD laboratory data. Furthermore, prescribing tinzaparin in HD patients who are of the male gender or diabetic could lead to lower serum TG levels, while prescribing it to patients who are older, nondiabetic, and male could have significant advantages in the control of serum TC.

In 1992, Akiba et al. compared the LMWH Logiparin and UFH in 33 HD patients during a 6-month period and found that UFH could exacerbate dyslipidemia in HD patients [22]. Several subsequent studies published within five years of this report also reported that LMWH can lead to a better lipid profile than UFH in chronic HD patients [6, 18, 19, 23]. However, some recent studies have observed no differences in the lipid profiles between these patient groups [12–14, 21, 24]. Therefore, it remains to be clarified as to whether LMWH can cause a better lipid profile than UFH in HD patients. Although our study was a cross-sectional observational one, the number of subjects was greater than those of prior studies. Moreover, the association between the better lipid profile (decreased serum TG and TC levels) and tinzaparin was noted after adjusting for possible confounding factors. Hence, our study supports a strong association between the lipid profile and tinzaparin use in HD patients.

Uremia patients belong to the highest risk group for cardiovascular disease with hyperlipidemia, which is a significant contributor to atherosclerosis [26, 27]. However, reverse epidemiology has been proposed for the lipid profile in HD patients. In particular, higher mortality was associated with low plasma TG values in HD patients [28], implying that malnutrition occurs in this type of patient and that the effect of malnutrition on mortality exceeded the protective effect of the better lipid profile [29, 30]. Moreover, the 4D (Die Deutsche Diabetes Dialyse) study [31], AURORA trial (A Study to Evaluate the Use of Rosuvastatin in Subjects on Regular Hemodialysis: An Assessment of Survival and Cardiovascular Events) [32], and SHARP (the Study of Heart and Renal Protection) [33] all disclosed no benefit of lipid-lowering agents on cardiac mortality and nonfatal myocardial infarction in HD patients who had DM and were older than 50 years of age. Two potential explanations had been proposed for these findings. One possibility is that the primary causes of atherosclerosis and cardiovascular disease in the HD population are oxidative stress, inflammation [34, 35], HDL deficiency [36], the dysfunction and accumulation of intermediate-density lipoproteins and chylomicron remnants, and the presence of small dense LDL [37], with the inhibition of cholesterol synthesis unable to correct these abnormalities. Another possibility is that hypertriglyceridemia and not LDL cholesterol is the major atherogenic factor in the HD population [27, 38].

In the present study, tinzaparin maintained the serum albumin level, indicating that no malnutrition was present. Moreover, patients who received tinzaparin had a significantly low serum TG level; therefore, tinzaparin could potentially be beneficial in the prevention of vascular atherosclerosis in HD patients. Moreover, a single-bolus dose of tinzaparin at the start of HD appears to be effective and safe [24, 39], enabling nursing care to be more convenient when performing HD. Therefore, tinzaparin can be considered as a clinically superior alternative to UFH in the maintenance of HD. Nevertheless, the benefit of tinzaparin on cardiovascular mortality still needs to be verified.

A study limitation is that the causation linking the serum lipid profile and tinzaparin cannot be inferred. However, the present data add to the growing body of evidence that tinzaparin administration can lead to a better lipid profile than UFH in patients with chronic HD. Another limitation is the sample size is relatively small. However, this concern may be trivial because a significant relationship between the serum lipid profile and tinzaparin was noted.

## 5. Conclusion

Our findings indicate a significant relationship between the lipid profile and heparin type, with tinzaparin associated with lower serum TG and TC levels in HD patients. Based on the subgroup analysis, male patients with DM can benefit from the effect of lowered TG, while male patients without DM may benefit from the effect of lowered TC level after tinzaparin use. However, a large-scale randomized control trial is still needed to determine the causation between the lipid profiles and tinzaparin.

## Figures and Tables

**Table 1:** Demographic and clinical data stratified according to the use of tinzaparin and unfractionated heparin.

Variables	All (*n* = 434)	UFH (*n* = 386)	Tinzaparin (*n* = 48)	*P* value
Age (y)	65.0 ± 13.2	65.4 ± 13.2	62.0 ± 13.0	0.95
Male (%)	210 (48.4%)	192 (49.7%)	18 (37.5%)	0.11
Body mass index (kg/m^2^)	22.6 ± 3.7	22.5 ± 3.7	23.0 ± 3.8	0.399
HD duration (months)	93.0 ± 68.8	88.1 ± 67.4	133 ± 66.7	<0.001
Artificial kidney				0.491
Polysulfone (%)	267 (61.5%)	234 (60.6%)	33 (68.7%)	
PMMA (%)	121 (27.9%)	111 (28.7%)	10 (20.8%)	
Cellulose citrate (%)	46 (10.6%)	41 (10.6%)	5 (10.4%)	
Diabetes mellitus (%)	177 (40.8%)	163 (42.2%)	14 (29.1%)	0.082
SBP (mmHg)	142.8 ± 25.9	142.8 ± 26.0	142.9 ± 24.5	0.984
DBP (mmHg)	80.9 ± 62.6	81.2 ± 66.1	78.38 ± 16.9	0.770
Lipid-lowering agent (%)	68 (15.7%)	58 (15%)	10 (20.8%)	0.290
Kt/V	1.39 ± 0.19	1.38 ± 0.2	1.4 ± 0.2	0.060
iPTH (pg/mL)	261 ± 304	249± 307	341 ± 260	0.053
Hemoglobin (g/dL)	10.3 ± 1.2	10.3 ± 1.3	10.5 ± 1.3	0.212
Albumin (g/dL)	4.2 ± 0.4	4.2 ± 0.4	4.2 ± 0.4	0.353
AST (IU/L)	20.1 ± 23.3	20.5 ± 24.6	17.2 ± 5.8	0.357
ALT (IU/L)	21.2 ± 31.0	21.5 ± 32.7	18.9 ± 9.8	0.584
Iron (*μ*g/dL)	65.3 ± 23.5	65.6 ± 23.3	58.4 ± 17.9	0.057
Ferritin (*μ*g/dL)	602 ± 468	621 ± 500	471 ± 237	0.044
TIBC (*μ*g/dL)	211 ± 41	210 ± 41	217 ± 40	0.219
Total-bilirubin (mg/dL)	0.32 ± 0.2	0.33 ± 0.26	0.27 ± 0.09	<0.001
Alk-p (mg/dL)	92.8 ± 71.4	93.4 ± 74.1	88.3 ± 43.6	0.640
Uric acid (mg/dL)	6.9 ± 1.3	6.8 ± 1.3	7.0 ± 1.2	0.274
Sodium (meq/L)	139.3 ± 3.2	139.1 ± 3.3	140.3 ± 2.8	0.017
Potassium (meq/L)	4.6 ± 0.7	4.6 ± 0.7	4.7 ± 0.6	0.515
Ionized calcium (mg/dL)	4.9 ± 0.5	4.9 ± 0.5	5.1 ± 0.5	0.023
Phosphate (mg/dL)	5.1 ± 1.4	5.1 ± 1.4	5.23 ± 1.1	0.459

Values for continuous variables are expressed as mean ± standard deviation; values for categorical variables are given as a number (percentage). UFH: unfractionated heparin; HD: hemodialysis; PMMA: polymethylmethacrylate; SBP: systolic blood pressure; DBP: diastolic blood pressure; iPTH: intact parathyroid hormone; AST: aspartate aminotransferase; ALT: alanine aminotransferase; TIBC: total iron-binding capacity; Alk-p: alkaline phosphatase.

**Table 2:** The linear regression mode for evaluating the relationship between independent variables and lipid profile in HD patients.

Variables	Triglyceride				Total cholesterol			
	Crude		Multivariable Forward stepwise regression		Crude		Multivariable Forward stepwise repression	
	*β* (CI 95%)^1^	*P* value	*β* (CI 95%)	*P* value	*β* (CI 95%)	*P* value	*β* (CI 95%)	*P* value
Tinzaparin use	−39.9 (−76.7, −3.0)	0.034	−40.8 (−75.1, −6.5)	0.020	−9.3 (−21.6, 3.0)	0.138	−13.0 (−24.5, −1.56)	0.026
Sex (male)	−13.1 (−36.3, 10.0)	0.265	−26.8 (−48.1, −5.4)	0.014	−12.8 (−20.4, −5.1)	0.001	−19.1 (−26.5, −11.8)	<0.001
Age (per year)	−0.52 (−1.40, 0.35)	0.240			−0.32 (−0.61, −0.02)	0.032		
Diabetes	35.1 (11.7, 58.5)	0.003			−11.6 (−19.4, −3.8)	0.004	−12.0 (−20.0, −4.0)	0.003
BMI (per kg/m^2^)	8.51 (5.51, 11.5)	<0.001	6.33 (3.36, 9.31)	<0.001	−0.12 (−1.16, 0.91)	0.819		
SBP (per mmHg)	0.47 (0.02, 0.91)	0.039	0.54 (0.12, 0.96)	0.012	0.03 (−1.11, 0.18)	0.680		
DBP (per mmHg)	0.05 (−0.13, 0.23)	0.576			−0.03 (−0.10, 0.02)	0.213		
Lipid-lowering agent	36.5 (4.81, 68.3)	0.024			−1.8 (−12.5, 8.7)	0.729		
HD vintage (per month)	−0.35 (−0.52, −0.18)	<0.001	−0.18 (−0.34, −0.01)	0.029	−0.03 (−0.09, 0.02)	0.240	−0.78 (−0.13, −0.01)	0.010
AK (versus polysulfone)								
PMMA	17.4 (−8.9, 43.9)	0.195			−0.93 (−9.79, 7.93)	0.837		
Cellulose citrate	−5.3 (−43.9, 33.2)	0.786			8.16 (−4.74, 21.0)	0.215		
KT/V (per unit)	−87.4 (−149.7, −25.1)	0.006			−1.71 (−22.7, 19.3)	0.873		
Albumin (per g/dL)	26.5 (−4.52, 57.5)	0.094			33.5 (23.6, 43.5)	<0.001	27.6 (17.2, 38.0)	<0.001
AST (per IU/L)	0.23 (−0.26, 0.73)	0.359			−0.09 (−0.26, 0.07)	0.257		
ALT (per IU/L)	0.21 (−0.16, 0.58)	0.261			−0.02 (−0.15, 0.09)	0.678		
Hemoglobin (per g/dL)	8.37 (−0.87, 17.6)	0.076			6.30 (3.25, 9.34)	<0.001	5.0 (1.98, 8.04)	0.001
T-bilirubin (per mg/dL)	−23.5 (−69.6, 22.5)	0.315			−16.0 (−31.4, −0.72)	0.040		
Ferritin (per *μ*g/dL)	0.006 (−0.01, 0.03)	0.651			−0.004 (−0.012, 0.005)	0.373		
Iron (per *μ*g/dL)	0.329 (−0.16, 0.82)	0.190			0.28 (0.11, 0.44)	0.001		
TIBC (per *μ*g/dL)	0.86 (0.59, 1.12)	<0.001	0.70 (0.44, 0.97)	<0.001	0.21 (0.11, 0.30)	<0.001	0.10 (0.01, 0.19)	0.028
iPTH (per pg/mL)	−0.05 (−0.08, −0.01)	0.008			0.005 (−0.008, 0.018)	0.445		
Alk-p (per mg/dL)	−0.11 (−0.27, 0.05)	0.181			−0.007 (−0.06, 0.04)	0.788		
Sodium (per meq/L)	−4.62 (−8.18, −1.05)	0.011	−3.77 (−7.04, −0.50)	0.024	0.27 (−0.92, 1.47)	0.650		
Potassium (per meq/L)	−6.05 (−23.5, 11.0)	0.496			7.54 (1.74, 13.3)	0.011		
iCa (per mg/dL)	−0.50 (−22.6, 21.6)	0.965			2.18 (−5.21, 9.58)	0.562		
Phosphate (per mg/dL)	−3.36 (−11.8, 5.12)	0.436	−14.0 (22.4, −5.6)	0.001	3.15 (0.32, 5.98)	0.029		
Uric acid (per mg/dL)	17.9 (9.39, 26.5)	<0.001	16.2 (7.6, 24.8)	<0.001	4.85 (1.96, 7.75)	0.001		

^1^ *β* is the regression coefficient. Abbreviations: SBP, systolic blood pressure; DBP, diastolic blood pressure, HD, hemodialysis; AST, aspartate aminotransferase; ALT, alanine aminotransferase; TIBC, total iron-binding capacity; iPTH, intact parathyroid hormone; Alk-p, alkaline phosphatase.

**Table 3:** The linear regression mode for evaluating the relationship between independent variables and lipid profile in HD patients.

Variables	LDL				HDL			
	Crude		Multivariable Forward stepwise regression		Crude		Multivariable Forward stepwise regression	
	*β* (CI 95%)^1^	*P* value	*β* (CI 95%)	*P* value	*β* (CI 95%)^1^	*P* value	*β* (CI 95%)	*P* value
Tinzaparin use	−6.21 (−16.9, 4.5)	0.256			4.8 (−0.9, 10.5)	0.099		
Sex (male)	−2.0 (−8.8, 4.6)	0.543	−6.80 (−13.2, −0.39)	0.038	−7.9 (−11.4, −4.3)	<0.001	−5.84 (−9.29, −2.32)	0.001
Age (per year)	−0.26 (−0.51, −0.005)	0.046			−0.32 (−0.16, 0.10)	0.649		
Diabetes	−6.7 (−13.5, 0.11)	0.054			−11.3 (−14.8, −7.8)	<0.001	−5.46 (−8.89, −2.03)	0.002
BMI (per kg/m^2^)	0.15 (−0.74, 1.06)	0.731			−1.66 (−2.12, −1.21)	<0.001	−1.47 (−1.92, −1.02)	<0.001
SBP (per mmHg)	−0.007 (−0.13, 0.12)	0.917			−0.04 (−0.11, 0.03)	0.260		
DBP (per mmHg)	−0.03 (−0.08, 0.02)	0.255			−0.01 (−0.04, 0.01)	0.195		
Lipid-lowering agent	−10.3 (−19.5, −1.1)	0.028	−12.1 (−20.8, −3.43)	0.006	−0.26 (−5.22, 4.69)	0.917		
HD vintage (per month)	−0.04 (−0.09, 0.003)	0.064			0.06 (0.03, 0.09)	<0.001		
AK (versus polysulfone)								
PMMA	−6.01 (−10.0, −1.93)	0.004	2.96 (−4.29, 10.2)	0.423	−0.84 (−8.53, 6.83)	0.828		
Cellulose citrate	−1.22 (−7.16, 4.72)	0.686	12.39 (1.85, 22.94)	0.021	9.43 (−1.75, 20.6)	0.098		
KT/V	−12.0 (−30.2, 6.1)	0.194			24.1 (14.6, 33.6)	<0.001	11.4 (1.45, 21.4)	0.025
Albumin (per g/dL)	26.5 (17.8, 35.2)	<0.001	23.7 (14.9, 32.6)	<0.001	8.9 (4.2, 13.7)	<0.001	10.4 (6.10, 14.71)	<0.001
AST (per IU/L)	−0.29 (−0.43, −0.14)	<0.001	−0.27 (−0.41, −0.13)	<0.001	−0.1 (−0.18, −0.03)	0.005		
ALT (per IU/L)	−0.16 (−0.27, −0.05)	0.002			−0.08 (−0.13, −0.02)	0.006		
Hemoglobin (per g/dL)	4.67 (2.01, 7.32)	0.001			1.04 (−0.39, 2.48)	0.153		
T-bilirubin (per mg/dL)	−22.6 (−35.9, −9.43)	0.001			−8.59 (−15.7, −1.47)	0.018		
Ferritin (per *μ*g/dL)	−0.01 (−0.01, −0.003)	0.006			−0.006 (−0.009, −0.002)	0.004	−0.007 (−0.010, −0.003)	<0.001
Iron (per *μ*g/dL)	0.21 (0.07, 0.35)	0.003	0.18 (0.04, 0.32)	0.009	0.03 (−0.046, 0.107)	0.431		
TIBC (per *μ*g/dL)	0.09 (0.011, 0.172)	0.025			−0.008 (−0.05, 0.03)	0.704		
iPTH (per pg/mL)	<0.001 (−0.01, 0.01)	0.987			0.01 (0.007, 0.019)	<0.001	0.008 (0.002, 0.013)	0.006
Alk-p (per mg/dL)	−0.03 (−0.08, 0.01)	0.164			0.02 (−0.003, 0.047)	0.085		
Sodium (per meq/L)	0.83 (−0.20, 1.87)	0.114			0.26 (−0.28, 0.82)	0.344		
Potassium (per meq/L)	5.78 (0.73, 10.8)	0.025			3.82 (1.13, 6.51)	0.005		
iCa (per mg/dL)	−3.16 (−9.58, 3.25)	0.333			5.38 (1.99, 8.78)	0.002		
Phosphate (per mg/dL)	2.29 (−0.16, 4.75)	0.068			1.61 (0.30, 2.92)	0.016		
Uric acid (per mg/dL)	2.6 (0.07, 5.13)	0.044			−0.67 (−2.03, 0,67)	0.326		

^1^ *β* is the regression coefficient. Abbreviations: SBP, systolic blood pressure; DBP, diastolic blood pressure, HD, hemodialysis; AST, aspartate aminotransferase; ALT, alanine aminotransferase; TIBC, total iron-binding capacity; iPTH, intact parathyroid hormone; Alk-p, alkaline phosphatase.
